# Improving college students’ fact-checking strategies through lateral reading instruction in a general education civics course

**DOI:** 10.1186/s41235-021-00291-4

**Published:** 2021-03-31

**Authors:** Jessica E. Brodsky, Patricia J. Brooks, Donna Scimeca, Ralitsa Todorova, Peter Galati, Michael Batson, Robert Grosso, Michael Matthews, Victor Miller, Michael Caulfield

**Affiliations:** 1 The Graduate Center, CUNY, 365 5th Ave, New York, NY 10016 USA; 2 The College of Staten Island, CUNY, 2800 Victory Blvd, Staten Island, NY 10314 USA; 3 Lehman College, CUNY, 250 Bedford Park Boulevard West, Bronx, NY 10468 USA; 4 Washington State University Vancouver, 14204 NE Salmon Creek Ave, Vancouver, WA 98686 USA

**Keywords:** Fact-checking instruction, Lateral reading, Media literacy, Wikipedia, College students

## Abstract

College students lack fact-checking skills, which may lead them to accept information at face value. We report findings from an institution participating in the Digital Polarization Initiative (DPI), a national effort to teach students lateral reading strategies used by expert fact-checkers to verify online information. Lateral reading requires users to leave the information (website) to find out whether someone has already fact-checked the claim, identify the original source, or learn more about the individuals or organizations making the claim. Instructor-matched sections of a general education civics course implemented the DPI curriculum (*N* = 136 students) or provided business-as-usual civics instruction (*N* = 94 students). At posttest, students in DPI sections were more likely to use lateral reading to fact-check and correctly evaluate the trustworthiness of information than controls. Aligning with the DPI’s emphasis on using Wikipedia to investigate sources, students in DPI sections reported greater use of Wikipedia at posttest than controls, but did not differ significantly in their trust of Wikipedia. In DPI sections, students who failed to read laterally at posttest reported higher trust of Wikipedia at pretest than students who read at least one problem laterally. Responsiveness to the curriculum was also linked to numbers of online assignments attempted, but unrelated to pretest media literacy knowledge, use of lateral reading, or self-reported use of lateral reading. Further research is needed to determine whether improvements in lateral reading are maintained over time and to explore other factors that might distinguish students whose skills improved after instruction from non-responders.

## Introduction

Young adults (ages 18–29 years) and individuals with at least some college education are the highest Internet users in the USA (Pew Research Center, 2019a). These groups are also most likely to use at least one social media site (Pew Research Center, 2019b). Despite their heavy Internet and social media use, college students rarely “read laterally” to evaluate the quality of the information they encounter online (McGrew et al., 2018). That is, students do not attempt to seek out the original sources of claims, research the people and/or organizations making the claims, or verify the accuracy of claims using fact-checking websites, online searches, or Wikipedia (Wineburg & McGrew, 2017).

The current study reports findings from one of eleven colleges and universities participating in the Digital Polarization Initiative (DPI), a national effort by the American Democracy Project of the American Association of State Colleges and Universities to teach college students information-verification strategies that rely on lateral reading for online research (American Democracy Project, n.d; Caulfield, 2017a). The DPI curriculum was implemented across multiple sections of a general education civics course, while other sections taught by the same instructors received the “business-as-usual” civics curriculum. We evaluated the impact of the DPI curriculum on students’ use of lateral reading to accurately assess the trustworthiness of online information, as well their use and trust of Wikipedia. We also examined factors that might influence whether students showed gains in response to the curriculum, such as their prior media literacy knowledge.

### How do fact-checkers assess the trustworthiness of online information?

Fact-checking refers to a process of verifying the accuracy of information. In journalism, this process occurs internally before publication as well as externally via articles evaluating the accuracy of publicly available information (Graves & Amazeen, 2019). Ethnographic research on the practices of professional fact-checkers found that fact-checking methodology involves five steps: “choosing claims to check, contacting the speaker, tracing false claims, dealing with experts, and showing your work” (Graves, 2017, p. 524). Interest in the cognitive processes and strategies of professional fact-checkers is not surprising in light of concerns about the rapid spread of false information (i.e., “fake news”) via social media platforms (Pennycook et al., 2018; Vosoughi et al., 2018), as well as the emergence of fact-checking organizations during the twenty-first century, especially in the USA (Amazeen, 2020).

When assessing the credibility of online information, professional fact-checkers first “take bearings” by reading laterally. This means that they “[leave] a website and [open] new tabs along the browser’s horizontal axis, drawing on the resources of the Internet to learn more about a site and its claims” (Wineburg & McGrew, 2018, p. 53). This practice allows them to quickly acquire background information about a source. When reading laterally, professional fact-checkers also practice “click restraint,” meaning that they review search engine results before selecting a result and rely on their “knowledge of digital sources, knowledge of how the Internet and searches are structured, and knowledge of strategies to make searching and navigating effective and efficient” (Wineburg & McGrew, 2018, p. 55). In contrast to professional fact-checkers, both historians and college students are unlikely to read laterally when evaluating online information (Wineburg & McGrew, 2017).

### How do college students assess the trustworthiness of online information?

How individuals assess the credibility of information has been studied across a variety of fields, including social psychology (e.g., work on persuasion), library and information science, communication studies, and literacy and discourse (see Brante & Strømsø, 2018 for a brief overview). When assessing the trustworthiness of online social and political information, college students tend to read vertically. This means that they look at features of the initial webpage for cues about the reliability of the information, such as its scientific presentation (e.g., presence of abstract and references), aesthetic appearance, domain name and logo, and the usefulness of the information (Brodsky et al., 2020; McGrew et al., 2018; Wineburg & McGrew, 2017; Wineburg et al., 2020). College students’ use of non-epistemic judgments (i.e., based on source features) rather than epistemic judgments (i.e., based on source credibility or corroboration with other sources) has also been observed in the context of selecting sources to answer a question and when ranking the reliability of sources (List et al., 2016; Wiley et al., 2009).

When provided with opportunities to verify information, adults (including college students) rarely engage in online searches and when they do, they usually stay on Google’s search results page (Donovan & Rapp, 2020). While looking for information, college students rely on the organization of search engine results and prior trust in specific brands (e.g., Google) for cues about the credibility of the information (Hargittai et al., 2010). Low search rates, superficial search behaviors, and reliance on cognitive heuristics (e.g., reputation, endorsement by others, alignment with expectations) may be indicative of a lack of ability or lack of motivation to engage in critically evaluating the credibility of online information. According to the dual processing model of credibility assessment, use of more effortful evaluation strategies depends on users’ knowledge and skills, as well as their motivation (Metzger, 2007; Metzger & Flanagin, 2015). Drawing on the heuristic-systematic model of information processing (Chen & Chaiken, 1999), Metzger and colleagues argue that the need for accuracy is one factor that motivates users to evaluate the credibility of information. Users are more likely to put effort into evaluating information whose accuracy is important to them. In cases where accuracy is less important, they are likely to use less effortful, more superficial strategies, if any strategies at all.

### Teaching college students to read laterally

The current study focuses on teaching college students to read laterally when assessing the trustworthiness of online information. However, a number of other approaches have already been used to foster students’ credibility evaluation knowledge and skills. Lateral reading contrasts with some of these approaches and complements others. For example, teaching students to quickly move away from the original content to consult other sources contrasts with checklist approaches that encourage close reading of the original content (Meola, 2004). One popular checklist approach is the CRAAP test, which provides an extensive list of questions for examining the currency, relevance, authority, accuracy, and purpose of online information (Blakeslee, 2004; Musgrove et al., 2018). On the other hand, lateral reading complements traditional sourcing interventions that teach students how to identify and leverage source information when assessing multiple documents (Brante & Strømsø, 2018). More specifically, lateral reading instruction emphasizes that students need to assemble a collection of documents in order to be able to assess information credibility, identify biases, and corroborate facts.

Lateral reading also aligns with aims of media, news, and information literacy instruction. Media literacy instruction teaches students how to access, analyze, evaluate, create, reflect, and act on media messages as means of both protecting and empowering them as media consumers and producers (Hobbs, 2010, 2017). Media literacy interventions can increase students’ awareness of factors that may affect the credibility of media messages, specifically that media content is created for a specific audience, is subject to bias and multiple interpretations, and does not always reflect reality (Hobbs & Jensen, 2009; Jeong et al., 2012). These media literacy concepts also apply in the context of news media (Maksl et al., 2017). Lateral reading offers a way for students to act on awareness and skepticism fostered through media and news literacy interventions by leaving the original messages in order to investigate sources and verify claims. While media and news literacy instruction focuses on students’ understanding of and interactions with media content, information literacy instruction teaches students how to search for and verify information online (Koltay, 2011). Being information literate includes understanding that authority is constructed and contextual and “us[ing] research tools and indicators of authority to determine the credibility of sources, understanding the elements that might temper this credibility” (Association of College & Research Libraries, 2015, p. 12). Lateral reading offers one means of investigating the authority of a source, including its potential biases (Faix & Fyn, 2020).

Lateral reading is also a necessary component of “civic online reasoning” during which students evaluate online social and political information by researching a source, assessing the quality of evidence, and verifying claims with other sources (McGrew et al., 2018). McGrew et al. (2019) conducted a pilot study of a brief in-class curriculum for teaching undergraduate students civic online reasoning. One session focused explicitly on teaching lateral reading to learn more about a source, while the second session focused on examining evidence and verifying claims. Civic online reasoning was assessed using performance-based assessments similar to those used in their 2018 study (McGrew et al., 2018). Students who received the curriculum were more likely to make modest gains in their use of civic online reasoning, as compared to a control group of students who did not receive the curriculum.

Aligning with this approach, the American Democracy Project of the American Association of State Colleges and Universities organized the Digital Polarization Initiative (DPI; American Democracy Project, n.d.) as a multi-institutional effort to teach college students how to read laterally to fact-check online information. Students were instructed to practice four fact-checking “moves”: (1) “look for trusted work” (search for other information on the topic from credible sources), (2) “find the original” (search for the original version of the information, particularly if it is a photograph), (3) “investigate the source” (research the source to learn more about its agenda and biases), and (4) “circle back” (be prepared to restart your search if you get stuck) (Caulfield, 2017a). Because emotionally arousing online content is more likely to be shared (Berger & Milkman, 2012), students were also taught to “check their emotions,” meaning that they should make a habit of fact-checking information that produces a strong emotional response.

In the current study, we were interested in fostering students’ use of lateral reading to accurately assess the trustworthiness of online content. Therefore, we focused specifically on students’ use of the first three fact-checking “moves.” These moves are all examples of lateral reading, as they require students to move away from original content and conduct searches in a new browser window (Wineburg & McGrew, 2017), and align with the practices of professional fact-checkers. While the DPI curriculum also taught the move of “circling back” and encouraged students to adopt the habit of “checking their emotions,” this move and habit are difficult to assess through performance-based measures and were not the focus of the assessments or analyses presented here.

## Research objectives

We present results from an efficacy study that used the American Democracy Project’s DPI curriculum to teach college students fact-checking strategies through lateral reading instruction. Students in several sections of a first-year, general education civics course received the DPI curriculum in-class and completed online assignments reinforcing key information and skills, while other sections received the “business-as-usual” civics instruction.

We were interested in whether students who received the DPI curriculum would be more likely to use lateral reading to correctly assess the trustworthiness of online content at posttest, as compared to “business-as-usual” controls. Additionally, we wanted to know the extent to which attempting the online assignments, which reviewed the lateral reading strategies and provided practice exercises, contributed to students’ improvement. As part of the analyses, we controlled for prior media literacy knowledge. Even though media literacy has not been tied directly to the ability to identify fake news (Jones-Jang et al., 2019), students with greater awareness of the media production process and skepticism of media coverage may be more motivated to investigate online content.

As part of the team implementing the DPI curriculum, we were provided with performance-based assessments like the ones used by McGrew et al. (2018) and McGrew et al. (2019) to assess students’ lateral reading at pretest and posttest. These types of assessments are especially critical given findings that college students’ self-reported information evaluation strategies are often unrelated to their observed behaviors (Brodsky et al., 2020; Hargittai et al., 2010; List & Alexander, 2018). In light of previous research on the disconnect between students’ self-reported and observed information-evaluation behaviors, we also examined whether students who received the DPI curriculum were more likely to self-report use of lateral reading at posttest, as compared to “business-as-usual” controls.

In the DPI curriculum, one of the sources that students are encouraged to consult when reading laterally is Wikipedia. Even though they are often told by secondary school teachers, librarians, and other college instructors that Wikipedia is an unreputable source (Garrison, 2018; Konieczny, 2016; Polk et al., 2015), students may rely on Wikipedia to acquire background information on a topic at the start of their searches (Head & Eisenberg, 2010). Therefore, we were interested in whether college students who received the DPI curriculum would report higher use of and trust of Wikipedia at posttest, as compared to “business-as-usual” controls.

Lastly, for students who received the DPI curriculum, we explored factors that might distinguish students who used lateral reading to correctly assess the trustworthiness of online content at posttest from their classmates who did not read laterally. In an effort to distinguish groups, we compared students on their use of lateral reading at pretest and their self-reported use of lateral reading at pretest. We also examined group differences in general media literacy knowledge at pretest, use of and trust of Wikipedia at pretest, and number of online homework assignments attempted.

## Methods

### Participants

First-year college students (*N* = 230) enrolled in a general education civics course at a large urban public university in the northeastern USA took part in the study. The university has an open-admission enrollment policy and is designated as a Hispanic-serving institution. Students took classes at main and satellite campuses, both serving mostly commuter students. Participants’ self-reported demographics are presented in Table 1. Almost half (47.8%) were first-generation students (i.e., neither of their parents attended college).

**Table 1:** Participants’ self-reported demographics for matched sections (*N* = 230; *N*_DPI_ = 136, *N*_Control_ = 94)

Characteristics	DPI	Control
Age		
Under 18	11.8	11.7
18–20	68.4	73.4
21–24	14.0	9.6
25–29	2.9	4.3
30–34	0.7	0.0
35–39	0.0	1.1
40–49	1.5	0.0
50 or older	0.7	0.0
Gender		
Female	58.1	47.9
Male	41.2	51.1
Another gender identity/prefer to self-describe	0.0	0.0
Prefer not to respond	0.7	1.1
Race/Ethnicity (not mutually exclusive)		
American Indian/Alaska Native	2.2	1.1
Asian/Asian American	19.1	10.6
Black/African-American	20.6	22.3
Latinx, Chicanx, Hispanic, or Spanish origin	22.8	26.6
Middle Eastern/North African	4.4	5.3
Native Hawaiian/Other Pacific Islander	0.0	0.0
White	31.6	40.4
Some other race	1.5	0.0
Prefer not to say	5.1	1.1
Unavailable/unknown	0.7	0.0
Either parent attended college		
Yes	49.3	56.4
No	50.7	43.6
Native English speaker		
Yes	75.0	81.9
No	25.0	18.1

Prior to the outset of the semester, the course instructors received training in the DPI curriculum and met regularly throughout the semester to go over lesson plans and ensure fidelity of instruction. Four instructors taught “matched” sections of the civics course, i.e., at least one section that received the DPI curriculum and at least one section that was a “business-as-usual” control. Two of the instructors taught one DPI section and one control section at the main campus, one instructor taught one DPI and one control section at the satellite campus, and one instructor taught one DPI and one control section at the main campus and one DPI section at the satellite campus. Across the matched sections, we had *N* = 136 students in the five DPI sections and *N* = 94 students in the four control sections. The research protocol was classified as exempt by the university’s institutional review board.

### The DPI curriculum

Students in DPI and control sections completed the online pretest in Week 3 and online posttest in Week 10 of a 15-week semester. The pretest and posttest were given as online assignments and were graded based on completion. For the pretest and posttest, materials were presented in the following order: lateral reading problem set, demographic questions, Wikipedia use and trust questions, self-reported use of lateral reading strategies, general media literacy scale, and language background questions. All materials are described below.

In the DPI sections, instructors spent three class sessions in Weeks 4 and 5 introducing students to the four fact-checking “moves” using two slide decks provided by developers of the DPI curriculum to colleges and universities participating in this American Democracy Project initiative. A script accompanying the slide decks guided instructors through explaining and demonstrating the moves to students. The slide decks included many examples of online content for instructors and students to practice fact-checking during class. The in-class DPI curriculum drew heavily on concepts and materials from Caulfield (2017a).

In the first slide deck, students were introduced to the curriculum as a way to help them determine the trustworthiness of online information. The four moves (look for trusted work, find the original, investigate the source, and circle back) were framed as “quick skills to help you verify and contextualize web content.” Students learned about the difference between vertical and lateral reading in the context of investigating the source. They also practiced applying three of the moves (looking for trusted work, finding the original, and investigating the source) to fact-check images, news stories, and blog posts by using the following techniques: checking Google News and fact-checking sites to find trusted coverage of a claim, using reverse image search to find the original version of an image, and adding Wikipedia to the end of a search term to investigate a source on Wikipedia.

In the second slide deck, students reviewed the three moves of looking for trusted work, finding the original, and investigating the source, as well as their associated techniques. Students were reminded that the fourth move, circle back, involved restarting the search if their current search was not productive. Students then learned that, in addition to using a reverse search to find the original version of an image, they could find the original source of an article by clicking on links. For investigating the source, students were told that they could also learn more about a source by looking for it in Google News. The remainder of the slide deck provided a variety of online content for students to practice fact-checking information using the four moves.

In Weeks 7 and 8, students in DPI sections spent three class sessions practicing evaluating online content related to immigration. This topic was chosen because it aligned with course coverage of social issues in the USA. Students were also given three online assignments to review and practice the strategies at home using online content related to immigration. These online assignments were graded based on completion and are described in detail below.

Aside from giving the pretest and posttest as online assignments, instructors in control sections followed the standard civics curriculum (i.e., “business as usual”), which focused on the US government, society, and economy, with no mention of lateral reading strategies and/or how to evaluate online content. As students in the control sections did not complete the three interim online homework assignments, the instructors implemented their regular course assignments, such as group projects.

Pretest, posttest, and online assignments were all administered via Qualtrics software with the links posted to the Blackboard learning management system. The script, slide decks, and online homework assignments are publicly available in an online repository.^1^

#### Lateral reading problems

Two sets of lateral reading problems (problem sets A and B) were provided by the developers of the DPI curriculum to all 11 campuses. Problems were adapted from the Stanford History Education Group’s civic online reasoning curriculum (Stanford History Education Group, n.d.) and from the Four Moves blog (Caulfield, 2017b). To ensure fidelity of implementation across campuses, we did not make any changes to the problem sets. Students completed one of the lateral reading problem sets (A or B) as a pretest and the other problem set as a posttest. Set order was counterbalanced across instructors: students in sections taught by two instructors received problem set A at pretest and problem set B at posttest, and students in sections taught by the other two instructors received problem set B at pretest and problem set A at posttest.

**Fig. 1:**
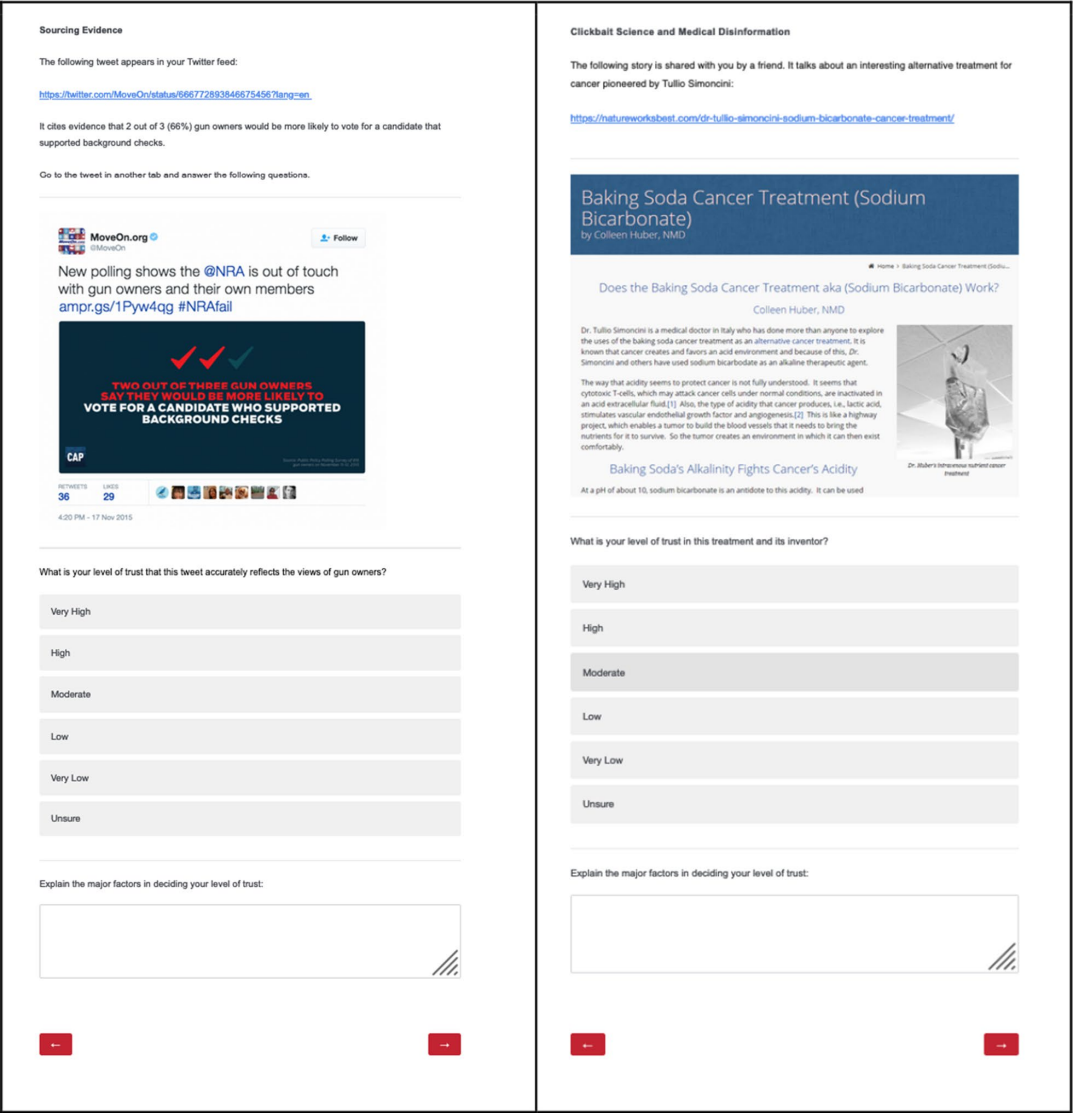
Screenshots of two of the lateral reading problems. *Note*: The left panel shows the Sourcing Evidence problem from problem set A, and the right panel shows the Clickbait Science and Medical Disinformation problem from problem set B

Each problem set consisted of one of each of four types of lateral reading problems determined by the developers of the DPI curriculum. The problems in each set included some problems with accurate online content, while other problems featured online content that was less trustworthy. Each problem was labeled by its problem type in order to frame the problem, but students could use multiple lateral reading strategies to fact-check each problem. For each problem, students indicated their level of trust in the online content using a Likert scale ranging from *1* = Very Low to *5* = Very High. Students could also indicate that they were Unsure (− *9*). Students were then prompted to “Explain the major factors in deciding your level of trust” using an open-response textbox. See Table 2 for a list of each problem type, problem set, online content used, and correct trust assessments and Fig. 1 for screenshots of two example problems.

**Table 2:** Problem type, online content, and correct trust assessment for problem sets A and B

Problem type	Problem set	Online content	Correct trust assessment
Photographic evidence	A	Photograph on Imgur claiming to show mutated flowers near the Fukushima Daiichi Nuclear Power Plant in Japan (https://imgur.com/gallery/BZWWx)	Low (1) Very Low (2)
	B	Photograph claiming to show Japanese Beetles attached to the roof of a dog’s mouth	Moderate (3) High (4) Very High (5)
Sourcing evidence	A	Tweet from MoveOn.org stating that “2 out of 3 gun owners would be more likely to vote for a candidate that supported background checks” (https://twitter.com/MoveOn/status/666772893846675456?lang=en)	Low (2) Moderate (3) High (4)
	B^a^	YouTube video from the National Mining Association titled “The Importance of Advanced Coal Technologies” (https://www.youtube.com/watch?v=vqLb0DkFOeI)	Low (2) Moderate (3) High (4)
Clickbait science and medical disinformation	A	Article published on BioNews titled “Majority of breast cancer patients do not need chemotherapy” (https://www.bionews.org.uk/page_136385)	Moderate (3) High (4) Very High (5)
	B	Article from the NatureWorksBest Cancer Clinic about the “Baking Soda Cancer Treatment (Sodium Bicarbonate)” (https://natureworksbest.com/dr-tullio-simoncini-sodium-bicarbonate-cancer-treatment/)	Low (1) Very Low (2)
Fake news	A	Article published on newser titled “School District Arms Students with Rocks” (http://www.newser.com/story/256977/school-district-arms-students-with-rocks.html)	Moderate (3) High (4) Very High (5)
	B	Article published on Big League Politics titled “Child’s Skull Found At Alleged Sex-Trafficking Bunker Area In Tucson” (https://bigleaguepolitics.com/breaking-childs-skull-found-at-alleged-sex-trafficking-bunker-in-tucson/)	Low (1) Very Low (2)

^a^The YouTube video used for the Sourcing Evidence problem in Set B at pretest was removed from YouTube after the pretest was administered. It was replaced with this video from the National Mining Association for the posttest

#### Scoring of lateral reading problems

The DPI provided a rubric for scoring student responses to the prompt “Explain the major factors in deciding your level of trust”: *0* = made no effort, *1* = reacted to or described original content, *2* = indicated investigative intent, but did not search laterally, *3* = conducted a lateral search using online resources such as search engines (e.g., Google), Wikipedia, or fact-checking sites (e.g., Snopes, PolitiFact) but failed to correctly evaluate the trustworthiness of the content (i.e., came to the incorrect conclusion or focused on researching an irrelevant aspect of the content to inform their decision), or *4* = conducted a lateral search and correctly evaluated the trustworthiness of the content. We established inter-rater reliability using the DPI’s rubric by having two authors independently score a randomly selected 16.5% of the responses for each lateral reading problem in each problem set.^2^ Since we used an ordinal scoring scheme ranging from *0* to *4*, we calculated weighted Cohen’s Kappa *k* = 0.93 as a measure of inter-rater agreement, which takes into account the closeness of ratings (Cohen, 1968). All disagreements were resolved through discussion. The authors then divided and independently coded the remaining responses.

Given the volume of responses, we decided to verify manual scores of *4* using an automated approach. First, we identified keywords that were indicative of use of lateral reading and searched each response for those keywords. Keywords were determined using a top-down and bottom-up approach, meaning that some words came from the curriculum, while other words were selected by scanning students’ responses. Table 3 presents keywords and sample responses for keywords. Responses that used at least one keyword were scored as *1*, indicating that the student read laterally. Responses that did not use any keywords were scored *0*, indicating that the student did not read laterally. Next, we scored responses on the Likert scale asking about the trustworthiness of the online content as *0* for incorrect trust assessment and *1* for correct trust assessment (see Table 2). Lastly, we combined the keyword and trust scores so that *0* indicated no use of lateral reading or use of lateral reading but with an incorrect trust assessment, and *1* indicated use of lateral reading with a correct trust assessment, which was equivalent to a manual score of *4*.

**Table 3:** Keywords used to automatically score responses for lateral reading

Type	Keywords	Sample response
Consulting external sources	wiki*, googl*, snope, politifact, cnn, breitbart, huffington, national geographic	“I looked up "Big League Politics" on wikipedia, but there was not a lot of information on it. I did find that it was founded by employees of Breitbart News, which was a conservative website that was described as racist and misogynistic. I also looked up the title of the article which led to a snopes page which said it was false. https://en.wikipedia.org/wiki/Breitbart_Newshttps://www.snopes.com/fact-check/was-childs-skull-found-alleged-sex-trafficking-bunker/”
Searching	revers*, search, searched, researched, researching, looked up, look up, looked for, look into, looking up, looked it up	“In order to decide whether to trust the photo or not, I reversed the image. I was able to fact check it on a website. The website mentioned that the flowers were not mutated due to radiation.”
Referencing the four moves	investigat*, original, other websites, other sites, four moves, four factors, fact check, hoax, debunk	“By investigating the source, I went to the article and took a few keywords and looked it up. I was able to fact check through the google search engine. I found other sources that spoke on the situation of the school shooting where the teachers and students were armed with rocks. There were other sources, such as the National Post and the abc.net.”

We next reviewed responses where manual and automated scores did not match (58 out of 1787 responses = 3.2%, Cohen’s Kappa *k* = 0.80).^3^ Twenty-three were false positives (i.e., had an automated score of *1* and a manual score of *3* or less), and 35 were false negatives (i.e., had an automated score of *0* and a manual score of *4*). In six of the false-negative responses, students expressed a trust assessment in their open-ended response that explicitly contradicted their trust assessment on the Likert scale. All disagreements were resolved in favor of the manual scoring.

#### Self-reported use of lateral reading strategies

Students used a 5-point Likert scale ranging from *1* = Never to *5* = Constantly to respond to the prompt “How frequently do you do the following when finding information online for school work?” for the three fact-checking moves requiring lateral reading and the habit of checking their emotions. Each move was described using layman’s terms in order to make it clear for students in control sections who were not exposed to the DPI curriculum. Look for trusted work was presented as “check the information with another source,” find the original was presented as “look for the original source of the information,” and investigate the source was presented as two items: “find out more about the author of the information” and “find out more about who publishes the website (like a company, organization, or government).” Check your emotions was presented as “consider how your emotions affect how you judge the information,” but was not included in analyses because it reflects a habit, rather than a lateral reading strategy. The four-item scale showed good internal consistency at pretest (*α* = .80).

#### Use of Wikipedia

Students were asked to respond to the question “How often do you use Wikipedia to check if you can trust information on the Internet?” using a 5-point Likert scale ranging from *1* = Never and *5* = Constantly.

#### Trust of Wikipedia

Students were asked to respond to the question “To what extent do you agree with the statement that ‘people should trust information on Wikipedia’?” using a 5-point Likert scale ranging from *1* = Strongly Disagree to *5* = Strongly Agree.

#### General media literacy knowledge scale

Students completed an 18-item scale (6 reverse-scored items) assessing general and news media literacy knowledge (adapted from Ashley et al., 2013, and Powers et al., 2018). For each statement, students indicated the extent to which they agreed or disagreed with the statement using a 5-point Likert scale ranging from *1* = Strongly Disagree to *5* = Strongly Agree. The 18-item scale showed adequate internal consistency at pretest (*α* = .76); reliability increased after removing an item with low item-rest correlation (–.08) (*α* = .80). The 17-item scale was used in analyses. An exploratory principal components analysis conducted using *IBM SPSS Statistics* (version 27) found four components with clustering primarily based on whether or not the item was reverse-scored.^4^ Therefore, we interpreted clustering based on reverse-coding to be a statistical artifact and treated the scale as unidimensional. See “Appendix” for students’ agreement on each item by condition at pretest.

To determine accuracy of students’ media literacy knowledge, scores were recoded such that scores of *1* through *3* were recoded as *0* (inaccurate) and scores of *4* and *5* were recoded as *1* (accurate). “Appendix” also reports accuracy on each item by condition at pretest.

#### Online homework assignments

Students in the DPI sections completed three online assignments to practice the lateral reading strategies covered in class. For each assignment, students were prompted to recall the four moves and a habit for reading laterally, saw slides and videos reviewing the four moves and a habit, and practiced using the four moves and a habit to investigate the validity of online content related to immigration, a topic covered in the civics course. Online content was selected from the Four Moves blog (Caulfield, 2017b). The first homework assignment asked students to investigate an article from City Journal magazine titled “The Illegal-Alien Crime Wave” (Caulfield, 2018c), the second assignment asked students to investigate a photograph that purported to show a child detained in a cage by US Immigration and Customs Enforcement (Caulfield, 2018b), and the last assignment asked students to investigate a Facebook post claiming that Border Patrol demanded that passengers on a Greyhound bus show proof of citizenship (Caulfield, 2018a). The online assignments are publicly available in an online repository.^5^

## Results

Results are organized by research questions. All analyses were run in *R* (version 3.6.2; R Core Team, 2018; RStudio Team, 2016).

### Preliminary analyses of lateral reading at pretest

Prior to conducting analyses to compare students who received the DPI curriculum with “business-as-usual” controls on lateral reading at posttest, we ran a series of preliminary analyses on the pretest data to assist us in formulating the models used to evaluate posttest performance.

We first examined whether students’ average scores on lateral reading problems differed by instructor or condition at pretest. For this set of analyses the dependent variable was each student’s average score across the four problems, as assessed via the DPI rubric (0 to 4). Students’ average scores at pretest did not differ significantly by condition (*M*_DPI_ = 1.21, *SD* = 0.35 and *M*_Control_ = 1.22, *SD* = 0.42; *t*(228) = 0.18, *p* = .855), see Table 4 for breakdown by problem and condition. A one-way between-group ANOVA with the instructor as the between-group variable and average score across the four problems as the dependent variable indicated that pretest performance did not differ by instructor (*F*(3, 226) = 1.47, *p* = .223, *η*_*p*_^*2*^ = 0.02).

**Table 4:** Mean score for students in each condition for each problem at pretest and posttest (*N* = 230; *N*_DPI_ = 136, *N*_Control_ = 94)

Problem type	Pretest		Posttest	
	DPI	Control	DPI	Control
Photographic evidence	1.21 (0.52) (*N* = 135)	1.41 (0.73) (*N* = 93)	2.13 (1.34) (*N* = 136)	1.09 (0.46) (*N* = 94)
Sourcing evidence	1.27 (0.60) (*N* = 135)	1.18 (0.51) (*N* = 92)	1.83 (1.08) (*N* = 106^a^)	1.27 (0.52) (*N* = 84^a^)
Clickbait science and medical disinformation	1.18 (0.53) (*N* = 136)	1.19 (0.65) (*N* = 93)	2.15 (1.28) (*N* = 136)	1.11 (0.35) (*N* = 92)
Fake news	1.19 (0.54) (*N* = 135)	1.12 (0.59) (*N* = 93)	2.67 (1.34) (*N* = 135)	1.13 (0.52) (*N* = 92)

Scores should be interpreted on a scale of *0* = made no effort, *1* = reacted to or described original content, *2* = indicated investigative intent, but did not search laterally, *3* = conducted a lateral search using online resources but failed to correctly evaluate trustworthiness, and *4* = conducted a lateral search and correctly evaluated trustworthiness

^a^Smaller *N*s for posttest Sourcing Evidence problem in problem set B at posttest are due to missing data or students’ responses stating that the YouTube video was unavailable

At the level of individual students, 7.0% of students received a score of *4* (i.e., read laterally and correctly assessed trustworthiness) for at least one problem at pretest (5.9% of students in the DPI sections and 8.5% in the control sections; see Table 5 for breakdown by problem type and condition). There was no significant difference across conditions, *X*^2^(1) = 0.26, *p* = .612, or instructor, Fisher’s exact test *p* = .603. Therefore, to evaluate the effectiveness of the DPI curriculum, we chose to examine differences in students’ scores only at posttest. For the posttest models, we created a control variable to indicate whether or not the student had engaged in lateral reading and drew the correct conclusion about the trustworthiness of the online content on one or more problems at pretest. We also included a control variable for the instructor to account for possible differences in the fidelity of implementation of the DPI curriculum.

**Table 5:** Percentage of students in each condition who received a score of 4 (i.e., read laterally and drew the correct conclusion about the trustworthiness of the online content) on each problem type at pretest and posttest (*N* = 230; *N*_DPI_ = 136, *N*_Control_ = 94)

Problem type	Pretest		Posttest	
	DPI	Control	DPI	Control
Photographic evidence	1.5% (*N* = 135)	3.2% (*N* = 93)	28.7% (*N* = 136)	1.1% (*N* = 94)
Sourcing evidence	1.5% (*N* = 135)	1.1% (*N* = 92)	14.2% (*N* = 106^a^)	1.2% (*N* = 84^a^)
Clickbait science and medical disinformation	2.2% (*N* = 136)	2.2% (*N* = 93)	24.3% (*N* = 136)	0.0% (*N* = 92)
Fake news	2.2% (*N* = 135)	2.2% (*N* = 93)	43.7% (*N* = 135)	1.1% (*N* = 92)

^a^ Smaller *N*s for posttest Sourcing Evidence problem in problem set B at posttest are due to missing data or students’ responses stating that the YouTube video was unavailable

We next examined whether problem sets A and B and the four types of problems were of equal difficulty at pretest. Students’ average score across the four problems did not differ significantly by problem set (*M*_set A_ = 1.25, SD = 0.38 and *M*_set B_ = 1.18, SD = 0.37; *t*(228) = 1.36, *p* = .175). To examine differences in scores by problem type, we conducted a one-way repeated-measures ANOVA with problem type as a within-subject variable and score as the dependent variable. With a Greenhouse–Geisser correction for lack of sphericity, there was a main effect of problem type, *F*(2.95, 657.48) = 2.66, *p* = .048, *η*_*p*_^2^ = .01. Post hoc tests with Tukey adjustment for multiple comparisons indicated that the Fake News problem type was harder than the Photo Evidence problem type (*p* = .040). All other problem types were of comparable difficulty. For each problem type, sets A and B were of comparable difficulty, except for the Sourcing Evidence problem type, where set A had an easier problem (*M* = 1.35, SD = 0.64) than set B (*M* = 1.10, SD = 0.43), *t*(218.28) = 3.55, *p* < .001. We retained problem type as a control variable in the posttest models. Problem set order was counterbalanced at the level of instructor and therefore fully confounded with instructor (see above); hence, we chose not to include problem set as a control variable in order to be able to retain instructor as a control variable in the posttest models.

### Differences in online homework attempts

Among students who received the DPI curriculum, 6.6% of students attempted no online homework assignments, 14.7% attempted one homework assignment, 44.1% attempted two assignments, and 34.6% attempted all three online homework assignments. On average, students in the DPI sections attempted 2.07 assignments (SD = 0.87). Given different rates of engagement with the assignments, we included the number of assignments attempted in the posttest models.

### Differences in general media literacy knowledge

Across both conditions, students demonstrated high general media literacy knowledge at pretest (*M*_agreement_ = 3.92, SD = 0.42; *M*_accuracy_ = 74.0%, SD = 20.5%). Students’ agreement as assessed via the Likert scale did not differ significantly by condition (*M*_DPI_ = 3.90, SD = 0.42 and *M*_Control_ = 3.95, SD = 0.43; *t*(228) = 0.80, *p* = .425). The accuracy of students’ knowledge also did not differ significantly by condition (*M*_DPI_ = 73.4%, SD = 20.3% and *M*_Control_ = 74.7%, SD = 20.7%; *t*(228) = 0.49, *p* = .624). See “Appendix” for mean agreement and accuracy per question at pretest by condition.

### Changes in lateral reading at posttest

At posttest, students in DPI sections had an average score of *M* = 2.22 (SD = 0.92) across the four problems and received a score of *4* on an average of 1.07 problems (SD = 1.07). In contrast, students in control sections had an average score of *M* = 1.15 (SD = 0.30) and received a score of 4 on an average of 0.03 problems (SD = 0.23).

To address our primary research question, we ran a mixed-effects ordinal logistic regression model with a logit link using the *clmm* function of the *ordinal* package (Christensen, 2019) in *R* (R Core Team, 2018; RStudio Team, 2016); see Table 6. For each posttest problem, our ordinal dependent variable was the student’s score on the 0–4 scale from the DPI rubric. We included an intercept-only random effect for students. Our fixed effects were media literacy knowledge at pretest, use of lateral reading to make a correct assessment at pretest, instructor, problem type, condition (DPI vs. control), and the number of online assignments attempted.

**Table 6:** Mixed-effects ordinal logistic regression model used to predict score for each problem on a scale of 0 to 4 (*N* = 230)

Predictor variables	*B* (SE)	Odds ratio (95% CI)	*z*	*X*^2^
Intercept for 0|1	− 2.90 (0.64)	0.05 (0.02, 0.19)	− 4.56***	–
Intercept for 1|2	4.15 (0.60)	63.71 (19.69, 206.11)	6.93***	–
Intercept for 2|3	5.14 (0.62)	170.27 (51.01, 568.39)	8.35***	–
Intercept for 3|4	5.86 (0.63)	351.19 (102.48, 1203.49)	9.33***	–
Media literacy accuracy at pretest	1.40 (0.60)	4.05 (1.26, 12.98)	2.35*	5.56*
Lateral reading at pretest (No = 0)	0.89 (0.46)	2.44 (1.00, 5.98)	1.96^†^	3.81^†^
Instructor (Instructor 1 = 0)^a^	–	–	–	10.72*
Instructor 2	0.19 (0.36)	1.21 (0.59, 2.45)	0.52	–
Instructor 3	0.39 (0.35)	1.48 (0.75, 2.91)	1.12	–
Instructor 4	1.07 (0.36)	2.93 (1.45, 5.90)	3.01**	–
Problem type (sourcing evidence = 0)^a^	–	–	–	21.54***
Clickbait science and medical disinformation	0.02 (0.23)	1.02 (0.66, 1.60)	0.11	–
Fake news	0.79 (0.23)	2.19 (1.40, 3.43)	3.45***	–
Photographic evidence	− 0.12 (0.23)	0.89 (0.56, 1.40)	− 0.50	–
Condition (Control = 0)	1.73 (0.45)	5.66 (2.34, 13.68)	3.85***	14.71***
Number of assignments attempted	0.48 (0.18)	1.62 (1.15, 2.29)	2.76**	7.63**

For instructor, post hoc comparisons with Tukey adjustment for multiple comparisons indicated that instructor 4’s students were more likely to score higher than instructor 1’s students (*p* = .014). The difference between instructor 4 and instructor 2’s students approached significance (*p* = .054). For problem type, post hoc comparisons with Tukey adjustment for multiple comparisons indicated that students were more likely to score higher on Fake News than Sourcing Evidence (*p* = .003), Clickbait Science and Medical Disinformation (*p* = .002), and Photo Evidence (*p* < .001)

^†^*p* < .06, *p < .05, **p < .01, ***p < .001

^a^Baselines set based on the lowest number of problems read laterally and correctly assessed at posttest

Overall, the full model with all fixed effects and the random effect of student fit significantly better than the null model with only the random effect of student (*X*^*2*^(10) = 137.46, *p* < .001). For each fixed effect, we compared the fit of the full model to the fit of the same model with the fixed effect excluded. This allowed us to determine whether including the fixed effect significantly improved model fit; see Table 6 for model comparisons. All control variables (i.e., media literacy knowledge at pretest, use of lateral reading to make a correct assessment at pretest, instructor, and problem type) significantly improved model fit or approached significance as predictors of students’ scores on lateral reading problems. Controlling for all other variables, students in the DPI sections were more likely to score higher on lateral reading problems than students in the control sections. Attempting more homework assignments was also significantly associated with higher scores.

Therefore, we dichotomized manual scores by recoding scores of *4* as *1* to indicate that the response provided evidence of lateral reading with a correct conclusion about the trustworthiness of the online content; all other scores were recoded as *0*. We then re-ran the model above with the dichotomized version of the dependent variable to see whether findings differed. For each posttest problem, our dependent variable indicated whether or not students received a score of *4*, i.e., whether they read laterally and also drew the correct conclusion about the trustworthiness of the online content. We used a mixed-effects logistic regression model with a binomial logit link using the *glmer* function of the *lme4* package (Bates et al., 2014) in *R* (R Core Team, 2018; RStudio Team, 2016); see Table 7.

**Table 7:** Mixed-effects logistic regression model used to predict use of lateral reading and correct trustworthiness conclusion on each problem (*N* = 230)

Predictor variables	*B* (SE)	Odds ratio (95% CI)	*z*	*X*^2^
Intercept	− 8.62 (1.19)	0.00 (0.00, 0.00)	− 7.24***	–
Media literacy accuracy at pretest	1.07 (0.80)	2.92 (0.61, 14.09)	1.33	1.83
Lateral reading at pretest (No = 0)	1.22 (0.61)	3.39 (1.02, 11.24)	1.99*	4.07*
Instructor (Instructor 1 = 0)^a^	–	–	–	10.50*
Instructor 2	0.68 (0.51)	1.98 (0.73, 5.37)	1.33	–
Instructor 3	0.22 (0.48)	1.25 (0.49, 3.18)	0.47	–
Instructor 4	1.41 (0.51)	4.10 (1.52, 11.08)	2.78**	–
Problem type (sourcing evidence = 0)^a^	–	–	–	35.60***
Clickbait science and medical disinformation	0.79 (0.38)	2.19 (1.03, 4.65)	2.05*	–
Fake news	2.00 (0.39)	7.40 (3.47, 15.77)	5.19***	–
Photographic evidence	1.13 (0.38)	3.09 (1.47, 6.50)	2.97**	–
Condition (Control = 0)	3.59 (0.84)	36.08 (7.02, 185.48)	4.29***	25.10***
Number of assignments attempted	0.59 (0.21)	1.81 (1.20, 2.72)	2.85**	8.54**

For instructor, post hoc comparisons with Tukey adjustment for multiple comparisons indicated that instructor 4’s students were more likely to read laterally and make a correct conclusion than instructor 1’s students (*p* = .028) and instructor 3’s students (*p* = .033). For problem type, post hoc comparisons with Tukey adjustment for multiple comparisons indicated that students were more likely to read laterally and make a correct conclusion on Fake News than Sourcing Evidence (*p* < .001), Clickbait Science and Medical Disinformation (*p* < .001), and Photo Evidence (*p* = .018). Students were also more likely to read laterally and correctly assess Photo Evidence than Sourcing Evidence (*p* = .016)

^†^*p* < .06, *p < .05, **p < .01, ***p < .001

^a^Baselines set based on the lowest number of problems read laterally and correctly assessed at posttest

Overall, the full model with all fixed effects and the random effect of student fit significantly better than the null model with only the random effect of student (*X*^2^(10) = 161.30, *p* < .001). For each fixed effect, we again compared the fit of the full model to the fit of the same model with the fixed effect excluded; see Table 7 for model comparisons. All control variables except media knowledge at pretest significantly improved model fit, indicating that they were significant predictors of scoring *4*, i.e., reading laterally and drawing a correct conclusion about trustworthiness. Controlling for all other variables, students in the DPI sections were significantly more likely to receive a score of *4* than students in the control sections. Students who attempted more homework assignments were also significantly more likely to score *4*.

### Changes in self-reported lateral reading at posttest

Descriptive statistics for students’ self-reported use of lateral reading strategies at pretest and posttest are presented in Table 8. At pretest, students in the control and DPI sections did not differ in the frequency with which they self-reported using lateral reading strategies when finding information online for school work, *t*(228) = –1.30, *p* = .196. On average, students at pretest reported using lateral reading strategies between Sometimes and Often.

**Table 8:** Descriptive statistics for self-reported use of lateral reading strategies by time and condition (*N* = 230; *N*_DPI_ = 136, *N*_Control_ = 94)

Strategy	Pretest		Posttest	
	DPI	Control	DPI	Control
Check the information with another source	3.82 (0.86)	3.70 (0.98) (*N* = 93)	3.81 (0.89)	3.62 (0.88) (*N* = 92)
Look for the original source of the information	3.62 (1.02)	3.57 (1.06)	3.57 (1.03)	3.53 (1.05)
Find out more about the author of the information	3.01 (1.22)	2.85 (1.11)	3.26 (1.14)	2.99 (1.26)
Find out more about who publishes the website (like a company, organization, or government)	3.01 (1.14) (*N* = 135)	2.76 (1.12)	3.53 (1.07)	2.97 (1.27)
Consider how your emotions affect how you judge the information^a^	2.75 (1.04)	2.57 (0.98)	2.99 (1.04)	2.68 ( 1.08)
Overall Mean (four items)	3.36 (0.84)	3.22 (0.84)	3.54 (0.83)	3.28 (0.92)

Items should be interpreted on a scale of *1* = Never to *5* = Constantly

^a^Item not included in analyses because it refers to a habit rather than a lateral reading strategy

To examine whether students who received the DPI curriculum were more likely to self-report use of lateral reading at posttest, as compared to controls, we conducted a 2 × 2 repeated-measures ANOVA with time (pretest vs. posttest) as a within-subject variable, condition (DPI vs. control) as a between-subject variable, and mean self-reported use of lateral reading as the dependent variable. There was a significant main effect of time, *F*(1, 228) = 4.67, *p* = .032, *η*_*p*_^2^ = 0.02, with students reporting higher use of lateral reading at posttest (*M* = 3.44, SD = 0.87) than at pretest (*M* = 3.30, *SD* = 0.84). There was also a significant main effect of condition, *F*(1, 228) = 4.13, *p* = .043, *η*_*p*_^*2*^ = 0.02, with students in the DPI sections reporting higher use of lateral reading (*M* = 3.45, *SD* = 0.84) than students in the control sections (*M* = 3.25, SD = 0.88). The interaction of time and condition was not significant, *F*(1, 228) = 1.06, *p* = .304, *η*_*p*_^2^ = 0.01.

### Changes in use of and trust of Wikipedia at posttest

Descriptive statistics for students’ use of and trust of Wikipedia at pretest and posttest are presented in Table 9. Since we used single items with ordinal scales to measure these variables, we used the nonparametric Wilcoxon–Mann Whitney test to compare students’ use and trust of Wikipedia across conditions at pretest and posttest (UCLA Statistical Consulting Group, n.d.).

**Table 9:** Percentage of students who indicated each response for use and trust of Wikipedia by time and condition (*N* = 230; *N*_DPI_ = 136, *N*_Control_ = 94)

	Pretest		Posttest	
	DPI (%)	Control (%)	DPI (%)	Control (%)
How often do you use Wikipedia to check whether you can trust information on the Internet?				
Never (1)	28.7	27.7	21.3	29.8
Rarely (2)	26.5	30.9	22.1	22.3
Sometimes (3)	31.6	34.0	36.8	40.4
Often (4)	11.0	6.4	15.4	7.4
Constantly (5)	2.2	1.1	4.4	0.0
To what extent do you agree with the statement that “people should trust information on Wikipedia”?				
Strongly disagree (1)	27.9	19.1	13.2	24.5
Disagree (2)	27.2	38.3	35.3	29.8
No opinion (3)	28.7	29.8	32.4	27.7
Agree (4)	12.5	12.8	18.4	18.1
Strongly agree (5)	3.7	0.0	0.7	0.0

For each question, each column sums to 100% with slight deviations due to rounding

At pretest, students in DPI sections did not differ from students in control sections in their responses to the question *How often do you use Wikipedia to check whether you can trust information on the Internet?*, *Median* = 2 (Rarely) for both conditions, *W* = 6135.5, *p* = .591. However, at posttest, students in DPI sections reported using Wikipedia more often to fact-check information (*Median* = 3, Sometimes) as compared to controls (*Median* = 2, Rarely), *W* = 5358.5, *p* = .030.

At pretest, students in DPI and control sections did not differ in their responses to the question *To what extent do you agree with the statement that “people should trust information on Wikipedia”? Median* = 2 (Disagree) for both conditions, *W* = 6492, *p* = .835. At posttest, students in DPI sections tended to report a higher level of trusting information on Wikipedia (*Median* = 3, No opinion) than students in the control sections (*Median* = 2, Disagree), but the difference in trust was not significant, *W* = 5753.5, *p* = .181.

### Individual differences in lateral reading for students in DPI sections

To better understand individual differences in students’ responses to the DPI curriculum, we compared students who scored *4* (i.e., used lateral reading and correctly assessed trustworthiness) on at least one problem at posttest (*n* = 83 or 61.0% of students in DPI sections) with their peers who did not receive a score of *4* on any of the lateral reading problems at posttest (*n* = 53 or 39.0% of students in DPI sections). We first looked at group differences on whether or not students read laterally and drew the correct conclusion about the trustworthiness of the online content on at least one problem at pretest and on their self-reported use of lateral reading at pretest. Groups did not differ in use of lateral reading on pretest problems or self-reported use of lateral reading at pretest.

Next, we examined whether groups differed in their general media literacy knowledge at pretest and their use and trust of Wikipedia at pretest. There was no difference between groups in general media literacy knowledge (agreement and accuracy) at pretest or in their use of Wikipedia at pretest. However, students in DPI sections who used lateral reading on at least one problem at posttest reported significantly lower trust of Wikipedia at pretest (*Median* = 2, Disagree) than students who failed to read laterally (*Median* = 3, No opinion, *W* = 2790, *p* = .006).

Lastly, we examined whether groups differed in the number of online homework assignments attempted. Students in DPI sections who used lateral reading on at least one problem at posttest attempted more online homework assignments (*M* = 2.23, SD = 0.83) than students who did not read laterally at posttest (*M* = 1.81, SD = 0.88, *t*(134) = –2.80, *p* = .006).

## Discussion

The current study examined the efficacy of the Digital Polarization Initiative’s (DPI) curriculum to teach students fact-checking strategies used by professional fact-checkers. In particular, we examined whether students in sections that administered the curriculum showed greater use of lateral reading at posttest than “business-as-usual” controls. We also examined whether conditions differed in self-reported use of lateral reading and use and trust of Wikipedia at posttest. Additionally, to explore possible individual differences in student responses to the curriculum, we examined whether use of lateral reading to correctly assess the trustworthiness of online content at pretest, self-reported use of lateral reading at pretest, general media literacy knowledge at pretest, use of and trust of Wikipedia at pretest, and number of online homework assignments attempted distinguished students who read laterally on at least one posttest problem from their classmates did not read laterally at posttest.

At posttest, students who received the DPI curriculum were more likely to read laterally and accurately assess the trustworthiness of online content, as compared to their peers in the control classes. Notably, there were no differences at pretest, as students almost universally lacked the skills prior to receiving the DPI curriculum. These findings are in keeping with previous work by McGrew et al. (2019), showing that targeted instruction in civic online reasoning (including lateral reading) can improve college students’ use of these skills. We also observed that the number of online assignments attempted was associated with use of lateral reading at posttest, with students in DPI sections who read laterally on at least one problem at posttest attempting more online homework assignments than students in DPI sections who failed to read laterally at posttest. This correlation suggests that time devoted to practicing the skills was helpful in consolidating them. However, we cannot confirm that the homework was the critical factor as students who were more diligent with their homework may also have had better in-class attendance and participation or better comprehension skills. Students who put more time or effort into the homework assignments may also have provided more written justifications on the posttest problems that could be scored using the DPI rubric (Bråten et al., 2018).

While 61.0% of students read and accurately assessed at least one problem after receiving the DPI curriculum, students rarely received a score of *4* on all four problems at posttest. This finding echoes previous research showing that, even when explicitly told that they can search online for information, adults, including college students, rarely do so (Donovan & Rapp, 2020). It is possible that students may have been more motivated to use lateral reading on certain problems based on their interest or how much they valued having accurate information on the topic (Metzger, 2007; Metzger & Flanagin, 2015). It is also possible that, for problems that produced a strong emotional response, students may have struggled to “check their emotions” sufficiently to read laterally and draw a correct conclusion about the trustworthiness of the online content (Berger & Milkman, 2012). Neither of these concerns would have emerged at pretest as students were almost uniformly unaware of lateral reading strategies.

Since the DPI curriculum was delivered in-class, students’ responsiveness to the DPI curriculum and their performance on the posttest may also have been affected by course-related factors. We observed an effect of instructors in the current study, which speaks to the importance of providing professional development and training for instructors teaching students lateral reading strategies. Another course-related factor that we could not account for was students’ attendance during class sessions when the curriculum was taught. Moving delivery of the DPI curriculum to an online format, e.g., by incorporating the instruction into the online homework assignments, may help ensure fidelity of implementation of the curriculum and facilitate better tracking of student participation and effort.

On average, students answered the majority (74.0%) of general media literacy knowledge items correctly at pretest. While general media literacy knowledge at pretest significantly predicted scores on the 0–4 scale at posttest, it was not a significant predictor of the dichotomized score distinguishing students who did and did not receive a score of *4* (i.e., those who did vs. did not use lateral reading to draw correct conclusions about the trustworthiness of the online content). Also, notably, students in DPI sections who received a score of *4* on at least one problem at posttest did not differ in their media literacy knowledge from students in DPI sections who never scored *4*. These findings suggest that understanding of persuasive intent and bias in media messages may have helped students recognize the need to investigate or assess the credibility of the information, but it was not sufficient to motivate them to use the fact-checking strategies to draw the correct conclusions. Traditional media literacy instruction may also be too focused on the media message, rather than on the media environment (Cohen, 2018). Students may benefit from instruction that fosters understanding of how their online behaviors and features of the Internet (e.g., use of algorithms to personalize search results) shape the specific media messages that appear in their information feeds. The need for additional instruction about the online information environment is also reflected in recent findings from Jones-Jang et al. (2019) documenting a significant association between information literacy knowledge (i.e., knowledge of how to find and evaluate online information) and the ability to identify fake news.

In addition to examining students’ performance on the lateral reading problems, we also asked students to self-report their use of lateral reading (e.g., by checking information with another source or finding out more about the author of the information). At pretest, students in both conditions reported using lateral reading strategies between Sometimes and Often, even though very few students in either condition demonstrated lateral reading on any of the pretest problems. Although students in the DPI sections self-reported greater use of lateral reading as compared to controls, the DPI students who read at least one problem laterally at posttest did not differ in their self-reported use of lateral reading strategies from DPI students who failed to read laterally at posttest. These findings align with the dissociation between students’ perceived and actual use of lateral reading skills observed in prior studies of students’ information evaluation strategies (Brodsky et al., 2020; Hargittai et al., 2010; List & Alexander, 2018). The observed dissociation may be due to students’ lack of awareness and monitoring of the strategies they use when evaluating online information (Kuhn, 1999). Instruction should aim to foster students’ metastrategic awareness, as this may improve both the accuracy of their self-reported use of lateral reading and their actual use of lateral reading.

Several other explanations for this dissociation are also possible. Some students may have accurately reported their use of lateral reading at posttest, but did not receive any scores of *4* on the lateral reading problems because their trustworthiness assessments were all incorrect. Alternatively, List and Alexander (2018) suggest that the dissociation between students’ self-reported and observed behaviors may be due to self-report measures reflecting students’ self-efficacy and attitudes toward these behaviors or their prior success in evaluating the credibility of information, rather than their actual engagement in the target behaviors. Overall, although performance-based measures may be more time-consuming and resource-intensive than self-report assessments (Hobbs, 2017; List & Alexander, 2018; McGrew et al., 2019), they are necessary for gaining insight into students’ actual fact-checking habits.

Despite the emphasis of the DPI curriculum on using Wikipedia to research sources and its popularity among professional fact-checkers (Wineburg & McGrew, 2017), students in the DPI sections only reported modestly higher Wikipedia use at posttest as compared to controls, and no difference in trust. Difficulties with changing students’ use and trust of Wikipedia may reflect influences of prior experiences with secondary school teachers, librarians, and college instructors who considered Wikipedia to be an unreliable source (Garrison, 2018; Konieczny, 2016; Polk et al., 2015). While McGrew et al. (2017) argue that students should be taught how to use Wikipedia “wisely,” for example, by using the references in a Wikipedia article as a jumping-off point for their lateral reading, this approach may require instructors teaching fact-checking skills to change their own perceptions of Wikipedia and familiarize themselves with how Wikipedia works. In future implementations, the DPI curriculum may benefit from incorporating strategies for conceptual change (Lucariello & Naff, 2010) to overcome instructors’ and students’ misconceptions about Wikipedia. Notably, our analysis of individual differences in response to the curriculum indicated that DPI students who demonstrated lateral reading at posttest were *less* trusting of information on Wikipedia at pretest than their peers who failed to use lateral reading at posttest. This unexpected result suggests that the lateral reading strategies were more memorable for DPI students who initially held more negative views about trusting information on Wikipedia, possibly because using Wikipedia as part of the DPI curriculum may have induced cognitive conflict which can foster conceptual change (Lucariello & Naff, 2010).

Looking ahead, additional research is needed to parse out individual differences in students’ responses to the DPI curriculum. Over a third of students did not read laterally on any of the problems at posttest, but this was unrelated to their use of lateral reading to correctly assess the trustworthiness of online content at pretest, their self-reported lateral reading at pretest or their self-reported use of Wikipedia at pretest to check whether information should be trusted. Given prior work on the roles of developmental and demographic variables, information literacy training, cognitive styles, and academic performance in children and adolescents’ awareness and practice of online information verification (Metzger et al., 2015), it may be fruitful to examine the role of these variables in predicting students’ responsiveness to lateral reading instruction. In addition, students’ reading comprehension and vocabulary knowledge should be taken into consideration as language abilities may impact students’ success in verifying online content (Brodsky et al., 2020). Future research also needs to examine the extent to which gains in lateral reading are maintained over time and whether students use the strategies for fact-checking information outside of the classroom context.

## Conclusion

The current study, conducted with a diverse sample of college students, examined the efficacy of the DPI curriculum in teaching students to fact-check online information by reading laterally. Compared to another study of college students’ online civic reasoning (McGrew et al., 2019), we used a larger sample and a more intensive curriculum to teach students these skills. Our findings indicate that the DPI curriculum increased students’ use of lateral reading to draw accurate assessments of the trustworthiness of online information. Our findings also indicate the need for performance-based assessments of information verification skills as we observed that students overestimate the extent to which they actually engaged in lateral reading. The modest gains that students made in Wikipedia use at posttest highlight an important challenge in teaching lateral reading as college students as well as instructors may hold misconceptions about the reliability of Wikipedia and ways to use it as an information source (Garrison, 2018; Konieczny, 2016). Lastly, the lack of relation between general media literacy knowledge and use of lateral reading to draw correct conclusions about trustworthiness of online information suggests that understanding and skepticism of media messages alone is not sufficient to motivate fact-checking. Instead, teaching lateral reading as part of general education courses can help prepare students for navigating today’s complex media landscape by offering them a new set of skills.

## Data Availability

The R Markdown file, analysis code, and instructional materials used in the current study are available in the Open Science Framework repository at https://osf.io/9rbkd/.

## Appendix

Percentage of students with accurate media literacy knowledge by item and condition at pretest (*N* = 230; *N*_DPI_ = 136, *N*_Control_ = 94).

**Table:**

Item	Agreement		Accuracy	
	DPI *M* (SD)	Control *M* (SD)	DPI *M* (SD)	Control *M* (SD)
A news story that has good pictures is less likely to get published. (reverse-scored)	2.77 (0.91)	2.64 (0.83)	45.6% (50.0)	41.5% (49.5)
People who advertise think very carefully about the people they want to buy their product	3.94 (0.99)	4.11 (0.91)	74.3% (43.9)	84.0% (36.8)
When you see something on the Internet the creator is trying to convince you to agree with their point of view	3.78 (0.76)	3.70 (0.80)	69.1% (46.4)	64.9% (48.0)
People are influenced by news whether they realize it or not	4.04 (0.81)	4.16 (0.79)	80.1% (40.0)	83.0% (37.8)
Two people might see the same news story and get different information from it	4.10 (0.81)	4.12 (0.82)	86.0% (34.8)	85.1% (35.8)
Photographs your friends post on social media are an accurate representation of what is going on in their life. (reverse-scored)	2.29 (1.03)	2.18 (0.99)	64.7% (48.0)	67.0% (47.3)
People pay less attention to news that fits with their beliefs than news that doesn’t. (reverse-scored)	3.08 (1.11)	3.11 (0.97)	32.4% (47.0)	26.6% (44.4)
Advertisements usually leave out a lot of important information	3.90 (0.90)	3.94 (0.88)	73.5% (44.3)	75.5% (43.2)
News makers select images and music to influence what people think	3.98 (0.79)	4.01 (0.71)	79.3% (40.7)	81.9% (38.7)
Sending a document or picture to one friend on the Internet means no one else will ever see it. (reverse-scored)	1.74 (0.80)	1.71 (0.88)	83.8% (37.0)	84.0% (36.8)
Individuals can find news sources that reflect their own political values	3.93 (0.77)	4.05 (0.68)	80.1% (40.0)	81.9% (38.7)
A reporter’s job is to tell the truth^a^	3.11 (1.20)	3.07 (1.20)	37.5% (48.6)	39.4% (49.1)
News companies choose stories based on what will attract the biggest audience	4.23 (0.80)	4.20 (0.85)	84.6% (36.3)	84.9% (36.0)
When you see something on the Internet you should always believe that it is true. (reverse-scored)	1.76 (0.92)	1.60 (0.69)	83.8% (37.0)	92.6% (26.4)
Two people may see the same movie or TV show and get very different ideas about it	4.40 (0.69)	4.31 (0.76)	92.6% (26.2)	91.5% (28.1)
News coverage of a political candidate does not influence people’s opinions. (reverse-scored)	2.13 1.00)	2.26 (0.97)	69.1% (46.4)	70.2% (46.0)
People are influenced by advertisements, whether they realize it or not	4.13 (0.79)	4.20 (0.73)	86.8% (34.0)	87.1% (33.7)
Movies and TV shows don’t usually show life like it really is	3.66 (1.01)	3.78 (0.96)	62.5% (48.6)	69.1% (46.4)
Overall Mean (17 items)	3.90 (0.42)	3.95 (0.43)	73.4% (20.3)	74.7% (20.7)

All agreement scores are on a scale of *1* = Strongly Disagree to *5* = Strongly Agree. Items were reverse-scored prior to calculating overall means and standard deviations

^a^Item removed due to low item-rest correlation

## Footnotes

1 https://osf.io/9rbkd/.

2 Only 13.5% of the responses for the Sourcing Evidence problem in Set B were scored due to missing data or responses stating that the YouTube video was unavailable.

3 Thirty-nine additional responses had clerical errors in the manual scoring that were corrected prior to reliability calculations. There were also 53 responses that were either missing data or that stated that the YouTube video was unavailable. These responses are not included in reliability calculations.

4 Given that we expected components to be correlated, we used a direct oblimin rotation with Kaiser normalization (Costello & Osborne, 2005). For the four components with eigenvalues greater than 1.00, seven non-reverse scored items clustered on the first component, four reverse-scored items clustered on the second component, two non-reverse scored items clustered on the third component, and one reverse-scored item clustered on the fourth component. Three items were below our criteria of .40 for the minimum factor loading (Stevens, 2002, as cited in Field, 2009).

5 https://osf.io/9rbkd/.
